# Antitumor Effect of Epigallocatechin Gallate and Vincristine in Mice with L5178Y Lymphoma

**DOI:** 10.3390/plants12213757

**Published:** 2023-11-02

**Authors:** Georgina Almaguer, Gustavo Almaguer-Vargas, Eva María Molina-Trinidad, Marco Antonio Becerril-Flores, Brenda Montejano, Eduardo Madrigal-Santillan, Alejandra Hernández-Ceruelos, Ana Hilda Figueroa-Gutiérrez, Ethoan Montejano, José Ramón Montejano-Rodríguez

**Affiliations:** 1 Institute of Health Sciences, Autonomous University of the State of Hidalgo, Ex-Hacienda de la Concepción, Tilcuautla 42183, Mexico; 2 Plant Breeding Department, Horticulture Institute, Chapingo Autonomous University, Federal Highway Mexico-Texcoco km 38.5, Chapingo, Texcoco 56230, Mexico; 3 Department of Pharmacy, Hospital San José, Santiago de Querétaro 76180, Mexico; 4 Interdisciplinary Professional Unit of Biotechnology IPN, National Polytechnic Institute, Av. Acueducto, La Laguna Ticoman, Gustavo A. Madero, Mexico City 07340, Mexico; ethoanmontejano@gmail.com

**Keywords:** EGCG, cancer, vincristine, p53, Bcl2

## Abstract

The main objective of research into new therapies is the search for more efficacy and fewer toxic effects in cancer treatments. On one hand, vincristine (VCR) is a chemotherapeutic used in different kinds of tumors. On the other hand, epigallocatechin gallate (EGCG) is a green tea metabolite that has shown an antineoplastic effect in diverse investigations, so the objective of this work is to evaluate the antitumor effects of the EGCG/VCR combination on tumor volume and survival. To achieve this objective, the solid model of lymphoma L5178Y was used in BALB/c mice with different doses of VCR, EGCG, and their combination allowed tumor growth and survival time recording. After tumor collection, measurements, and immunohistochemistry for p53, Bcl2, and Cyclin D1 were performed. The results showed that the EGCG/vincristine combination had a greater antitumor effect than those effects of vincristine and EGCG. It can be attributed to the fact that the greatest inhibition of Bcl2 was present in gathering of EGCG harvest with vincristine. Therefore, the combination of EGCG with vincristine has a better antineoplastic effect by inhibiting tumor development and increasing survival on both substances independently.

## 1. Introduction

Cancer is a group of diseases caused by various alterations in oncogenes and suppressor genes, causing tumors associated with oncogenic signaling pathways [1], its high morbidity and mortality impact globally in an important way, both socially and economically. It is known that there is a wide range of therapies; however, its relative effectiveness and its various adverse reactions mean that concomitant therapies to current chemotherapy treatment continue to be sought in order to improve the therapeutic effect, reduce doses, reduce toxicity and improve the patient’s quality of life. Among these chemotherapies is vincristine, currently its clinical use is limited by its serious side effects observed in the cardiovascular system, the central nervous system, dermatological, endocrine, and metabolic systems, gastrointestinal and genitourinary systems, it causes hematological and oncological problems, it is derived from vinca is a monoterpene indole alkaloid. The main mechanism of action of VCR is by blocking cell growth and mitosis, preventing the formation of the mitotic spindle, acting mainly in the G2 phase [2,3].

Some plants have antitumor active ingredients that can create a window of opportunity for cancer patients whose economic conditions do not allow them to obtain affordable treatments, patients in whom existing treatments are not effective or generate too many adverse reactions and generally to improve the quality of life while the disease-therapy process. During the implementation of phytotherapy, green tea has attracted worldwide attention, its production and consumption have increased each year. It estimates that the production of green tea will increase by 7.5% per year to reach 3.6 million tons in 2027. One of the reasons for this increase is the attributed therapeutic qualities [4]. Among its secondary metabolites are polyphenols such as flavan-3-ols, which represent up to 30% of the dry weight. The one found in greatest abundance is (–)-Epigallocatechin gallate (EGCG).

**Figure 1:**
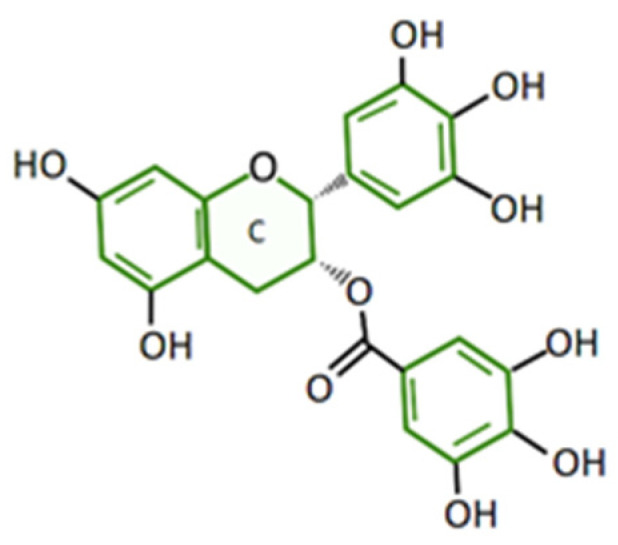
EGCG structure comprises four rings joined by the C pyran ring. Based on Du, G.-J., et al. 2012 [5].

Its structure comprises four aromatic rings joined by the C ring, which is a pyran, giving the molecule a concave shape. Figure 1.

It is considered responsible for a large part of the benefits provided by green tea. This metabolite is found in strawberries, apples, and cocoa, among others, and it is highly commercialized in green tea making EGCG a very important, highly consumed, and affordable phytochemical worldwide. It has shown that among its most striking properties are antioxidants, cardiovascular protection, antimutagenic, antiviral, and anticancer [5], validating this last effect as an antineoplastic, in vitro, and in vivo investigations have been carried out in different cancer models, for example, that of the pancreas [6], lung [7], breast [8,9], liver [10], and ovary [11]. Its toxicity has been extensively studied, this is because hepatoxicity has been reported in 10% of the population that consumed ≥800 mg of EGCG/day for ≥4 months, positively it is mentioned that the intake of 316 mg/day does not show elevated levels of liver damage enzymes, even suggesting that ingesting 5 or more cups of traditional infused green tea (approximately 700 mg) of EGCG per day does not increase transaminases [12].

Its low toxicity, and its most studied mechanisms of action against cancer are arrest of the cell cycle in the G1 phase and apoptosis, both by intrinsic and extrinsic routes [13,14], make it a candidate to study it as an adjuvant of VCR, since its effects can add to the antimitotic and apoptotic effects of vincristine, which acts mainly in the G2 phase of the cell cycle, also considering that the toxicity of both does not overlap.

The problem so far with its use in prevention or treatment at the clinical level is that the data obtained in humans and in vivo models have a lot of variability and are inconsistent and incompatible with the in vitro results [15], which has led to a conflict to advance towards clinical trials [16].

It is necessary to provide more evidence of the in vivo antitumor effect of EGCG alone or in combination to obtain better treatment efficacy and reduce adverse reactions or resistance to chemotherapy. This work team is aware of the importance of investigating the antitumor effect of EGCG alone or in combination, observing its effect in vivo alone and combined with vincristine sulfate in a solid murine lymphoma model.

## 2. Results

### 2.1. Effect of Different Treatments on Tumor Growth Inhibition

Quantifying the tumor volume to determine the effect of EGCG and its combination with vincristine in tumor growth inhibition.

**Figure 2:**
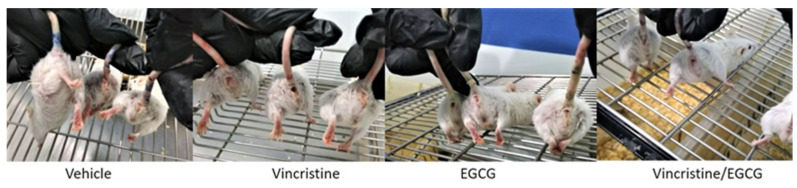
Caudal view of the groups treated with Vehicle, VCR, EGCG and concomitant treatment, greater adverse effects are observed in the vehicle and VCR groups such as shaggy hair, bedsores, poor mobility, and edema of the left hind limb.

The administration of the different treatments modifies the tumor size in the groups, causing differences that impacted the quality of life and death. The body weight gain of the animals corresponds to the increase in tumor growth; For example, the animals that showed the greatest weight gain were the control group, followed by VCR and subsequently the groups in which EGCG was administered, including the EGCG/VCR combination. Therefore, said weight gain obtained in the different groups cannot be attributed or interpreted as a gain due to decreased toxicity. However, the state of well-being observed in the groups subjected to the different treatments did show differences; on day 10 of the volume determination experiment, the following signs could be seen in the experimental groups:

(1) The control group presented rapid tumor growth, the left lower extremity where the tumor was inoculated showed a claw shape and was edematous. The appearance of the individuals in this group was of rough, bristly, sparse, and dirty fur: they presented isolated ulcers of up to half a centimeter, areas with scabs and red-dark skin, alopecia areas mainly in the ventral/dorsal caudal area, the perianal was dirty, the animals were stressed, they attacked each other, injuring their caudal extremity.
(2) Regarding the group with VCR, tumor growth, pain in the area of application of the medication, the left hind extremity without mobility, a red caudal area, hirsute hair and alopecia areas were observed. They also showed stress and aggression in the group.
(3) In both the group with EGCG and the group with the combination of EGCG and VCR, a decrease in tumor growth was observed, the individuals supported the limb where the tumor was located, they controlled the movement better, the hair showed a smooth appearance, they had no ulcerations; the skin had a pinkish hue and they did not present alopecia; However, there were injuries due to aggression among them. Figure 2.

#### 2.1.1. Effect of (–)-EGCG on the Inhibition of Tumor Growth

**Figure 3:**
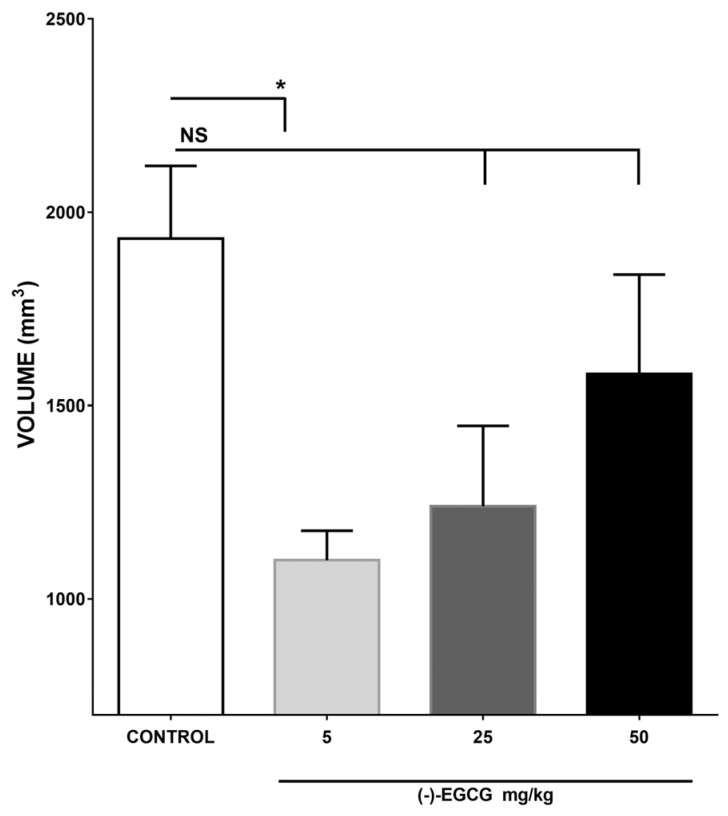
Effect of EGCG on L5178Y solid tumor volume. The figure shows the tumor volume of the vehicle group and different doses of EGCG (5, 25, 50 mg/kg). The tumors EGCG (5 mg/kg) groups showed inhibition in tumor growth compared with the control group, since while the control group presents an average of 1932.15 mm^3^, the group with EGCG at this dose shows a volume of 1099 mm^3^ (*p* < 0.01). Differently, in relation to the highest doses, that is, the groups with 25 and 50 mg/kg of the flavonoid were similar to the control by presenting means of 1239 mm^3^ and 1582 mm^3^ respectively, without a significant difference. NS without significant difference compared to the control group and * Significant statistical difference compared to the control group. Each bar represents the mean ± SE. n = 5. The analysis was implemented with the statistical test of analysis of variance (ANOVA), for the multiple comparison, Tukey was used. Values of *p* < 0.05 were considered statistically significant.

The comparison of the effect of the control group against the increasing doses of EGCG used 5, 25, and 50 mg/kg, showed that the reduction in tumor size was significant with the lowest doses used of catechin with 1099 mm^3^ concerning vehicle 1932.15 mm^3^ (*p* < 0.05). This effect represents a growth inhibition of 43.08% about the vehicle, without finding significance in the effects of the other doses used as shown in Figure 3.

#### 2.1.2. The Effect of Vincristine, and Its Combination with EGCG in the Inhibition of Tumor Growth

**Figure 4:**
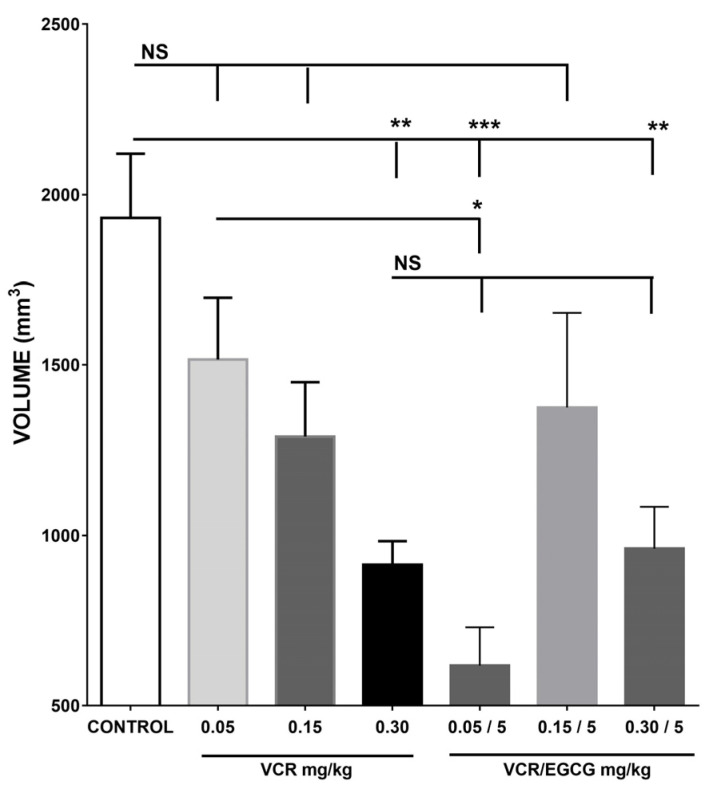
Tumor volume with vincristine and its combination with EGCG. The tumor volume (mm^3^) obtained 11 days after IM administration of 1 × 10^6^ L5178Y cells in BALB/c mice treated with VCR, showed a difference between the control group and the dose of 0.30 mg/kg/c7 days (*p* < 0.01), it was contrary to what was observed with the doses of 0.05 mg/kg and 0.15 mg/kg which is like the control. When administered concomitantly with VCR 0.05/EGCG 5.0 (mg/kg), there was a significant reduction in tumor volume against the control group (*p* < 0.001) with VCR 0.05 (*p* ≤ 0.05), without observing the same effect with the other doses used. NS is not No Statistical difference; *, ** and *** represent statistical difference of *p* ≤ 0.05, *p* ≤ 0.01 and *p* ≤ 0.001 respectively. Each bar represents the mean ± SE with n = 5. The data were analyzed by the statistical analysis of variance test (ANOVA) followed by the Tukey multiple comparison test. Values of *p* < 0.05 were considered statistically significant.

In the results with vincristine (VCR) was found that when using 0.05, 0.15, and 0.30 mg/kg only in the highest dose, less tumor growth was observed compared with the control group, obtaining a mean of 931.45 mm^3^ (*p* = 0.001). The values obtained with the VCR doses of 0.15 and 0.05 mg/kg were 1290 mm and 1516 mm, as observed in Figure 4.

Subsequently, the contrasting effect of administering different doses of CVR with 5 mg/kg EGCG was observed. This dose was selected because it was the one observed with tumor inhibition. Results showed a lower tumor volume when administering the doses of 0.05 mg/kg of VCR with 5.0 mg/kg of EGCG, with the control and the alkaloid, representing only 31.99% of the average volume of the vehicle group (618 mm^3^ against 1932 mm^3^ of the vehicle group) and 40.76% the average size in the vincristine group at this dose. However, with the other doses of vincristine used, the same effect was not observed. In other words, the combination with a higher dose of vincristine had no better effect than when using the chemotherapy alone, observing the same inhibition produced by any of them separately as show in Figure 4. On the other hand, it is important to point out that when comparing the effect in the groups with vincristine 0.30 mg/kg and catechin EGCG 5 mg/kg/day, the similarity was observed with tumor growth inhibition. As a result, EGCG exerted the same effect as VCR at this dose. The comparison of the effect of the control group against the increasing doses of EGCG used 5, 25, and 50 mg/kg, showed a tumor reduction.

### 2.2. Effect of EGCG, VCR, and Their Concomitant Administration on Survival

**Figure 5:**
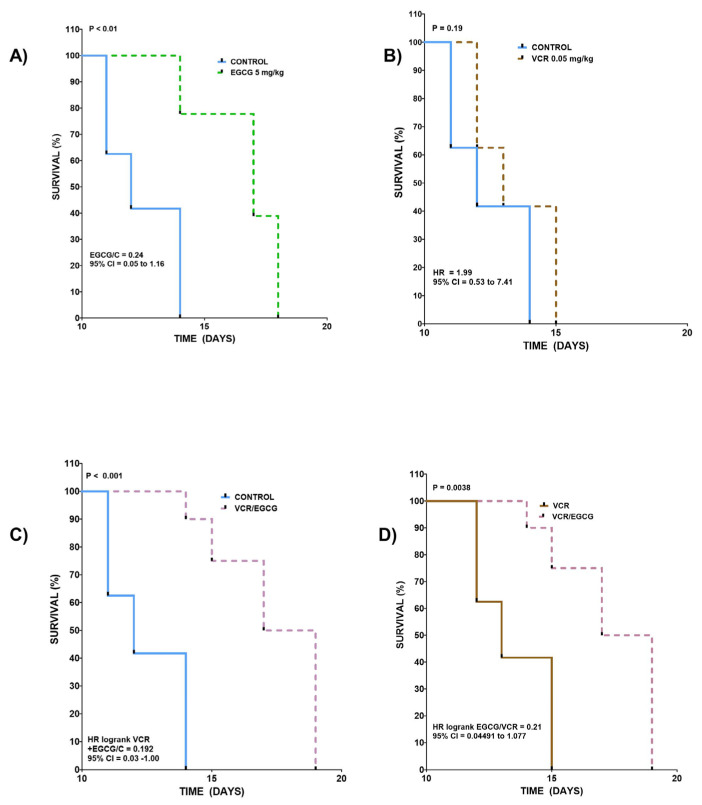
Survival curves with EGCG, VCR, and VCR/EGCG in L5178Y tumor. The survival curves of mice inoculated with L5178Y cells in the gastrocnemius muscle are show: (**A**) The curves of the vehicle group and EGCG 5 mg/kg are compared with medians of 12 and 17 days, showed a difference significant of *p* < 0.01; (**B**) Vehicle and VCR 0.05 mg/kg. The continuous line corresponds to the control group with a median of 12 days, and vincristine 0.05 mg/kg, represented by the dotted line, obtained a median of 13 days, like the control group. No significant difference was observed between the two groups. (*p* = 0.19); (**C**) Shows control and the combination of VCR/EGCG with a higher probability of survival in the group with the combination of VCR/EGCG than with the combination (*p* < 0.001; (**D**) The VCR curves and the VCR/EGCG combination are compared, the latter group represented by a dotted line, the curves present a median of 13 and 18 days respectively, showing a *p* = 0.0038, which can be interpreted as a possibility of greater 28% survival for combination than for VCR alone. Kaplan–Meier curves. (Log-rank). n ≥ 5. Values of *p* < 0.05 were considered statistically significant.

To determine the risk of death between the different groups, the survival curves were compared, which can be observed in Figure 5A. The results of the vehicle and EGCG (5 mg/kg) groups are graphed. The curves show medians of 12 and 17 days, with a shift to the right with the treated group. The ratio is 0.70 with 95% CI from 0.050 to 1.16, being different between them with a *p* < 0.01. The above allows us to say that in this model, the group with EGCG at this dose has a greater chance of survival, as seen in Figure 5B. This plots the vehicle group against VCR 0.05 mg/kg, with this dose, the curves and medians are similar (12 and 13 respectively) and intersect at different points, the Ratio being 1.99 is very close to 1, so no difference is observed between the two (*p* = 0.19), the 95% CI ranges from 0.53 to 7.41. These results are interpreted as giving this dose of VCR, or the vehicle provides the same chance of death possibility in both groups in the same period of time as seen in in Figure 5C. When analyzing the vehicle group against the curve of the group that received the combination, a shift to the right was observed since the medians were 12 and 18, respectively, a favorable ratio of 0.192 and 95% CI of 0.03 to 1 was observed. *p* = 0.001 so the curves are considered different in Figure 5D. When comparing the VCR curve (0.05 mg/kg) with the curve made with the VCR/EGCG combination, it was found that the medians were 13 days for VCR versus 18 for the combination, and the ratio was 0.21 with 95% CI of 0.044 to 1.077 showing a *p* = 0.0038, which can be interpreted as a greater chance of survival for the combination than for VCR alone [17,18].

### 2.3. Effect of EGCG, VCR, and VCR/EGCG on the Determination of p53, Cyclin D1 and Bcl2 in Murine Lymphoma

**Figure 6:**
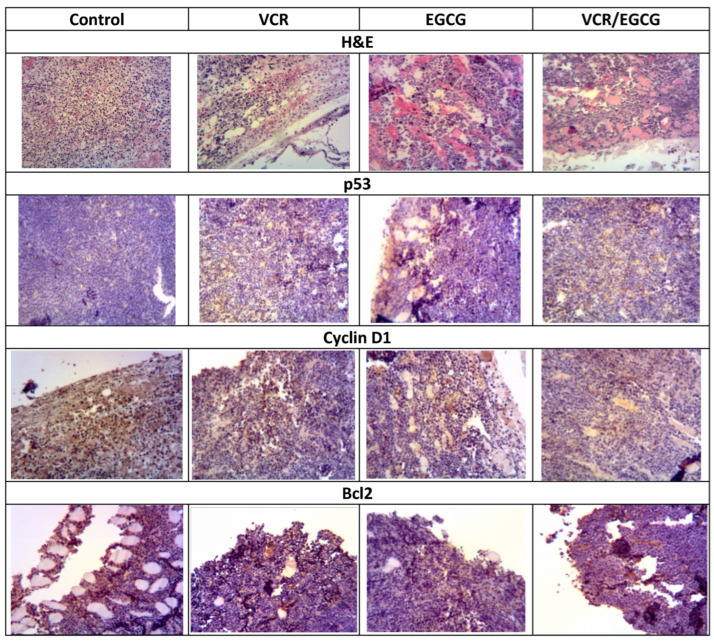
The figure presents microphotographs of the histology of the tumor with eosin-hematoxylin staining and with the immunohistochemistry of the different p53, Cyclin D1 and Bcl2 proteins of the groups treated with EGCG, VCR, and concomitant treatment.

The inhibition in tumor growth and the increase in survival prognosis led to the evaluation of some proteins involved in tumor development for which the presence of p53, Cyclin D1, and Bcl2 was analyzed by immunohistochemistry as seen in Figure 6.

**Figure 7:**
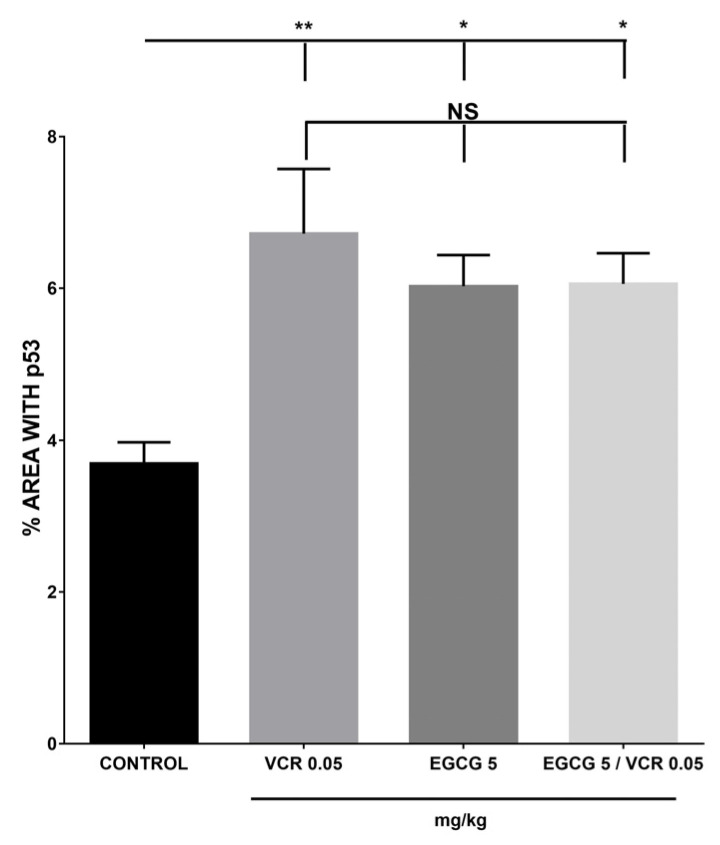
Determination of p53 with VCR, EGCG, and their adjuvant treatment in murine lymphoma. The percentage of p53 protein immunopositivity in murine lymphoma tissue is expressed, showing an increase with the different groups in relation to the control (*p* ≤ 0.05), with no statistical difference observed between the different groups with treatment. NS is equivalent to no statistical difference; * and ** represents statistical difference of *p* ≤ 0.05 and *p* ≤ 0.01 respectively. Each bar corresponds to the mean ± SE with n = 10. The data were analyzed by the analysis of variance (ANOVA) statistical test followed by the Tukey multiple comparison test. Values of *p* ≤ 0.05 were considered statistically significant.

For p53, the control group showed the lowest value of 3.69%, while the different treatments increased their presence, obtaining 6.92% with VCR and 5.99% with EGCG, respectively, the co-administration of both treatments presented 6.18%, being similar between all of them and different from the control (*p* < 0.05), as shown in Figure 7.

**Figure 8:**
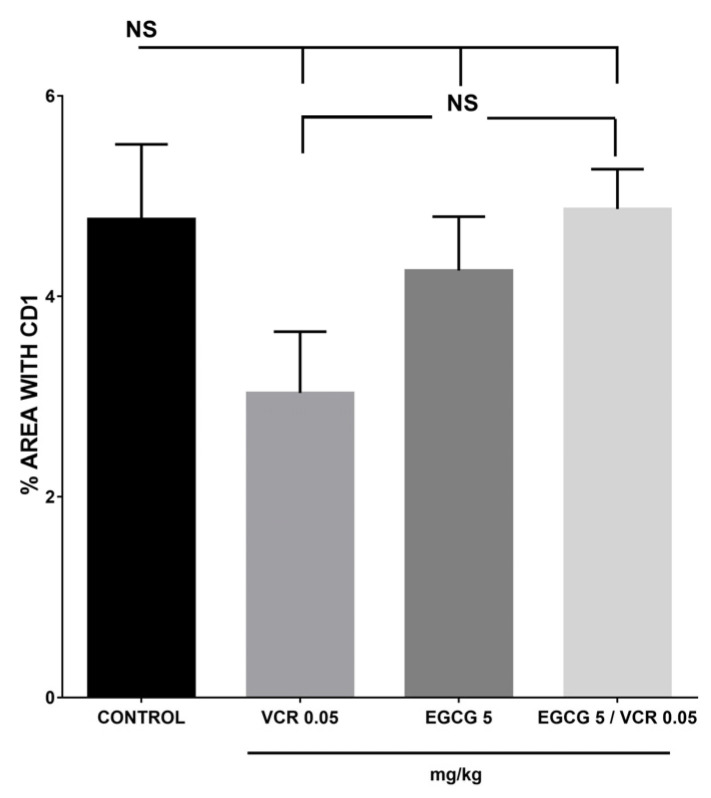
Determination of Cyclin D1 protein in lymphoma murine with EGCG, VCR, and VCR/EGCG. The presence of Cyclin D1 was positive in the tumor, both the control group and the different treatments were statistically similar. NS equals non-statistical significance. Each bar represents the mean ± SE with n = 10. The data were analyzed by the analysis of variance (ANOVA) statistical test followed by the Tukey multiple comparison test. Values of *p* < 0.05 were considered statistically significant.

The results obtained with the Cyclin D1 protein showed that the effect between the diverse treatments and the control was not significant *p* ≥ 0.05. Figure 8.

**Figure 9:**
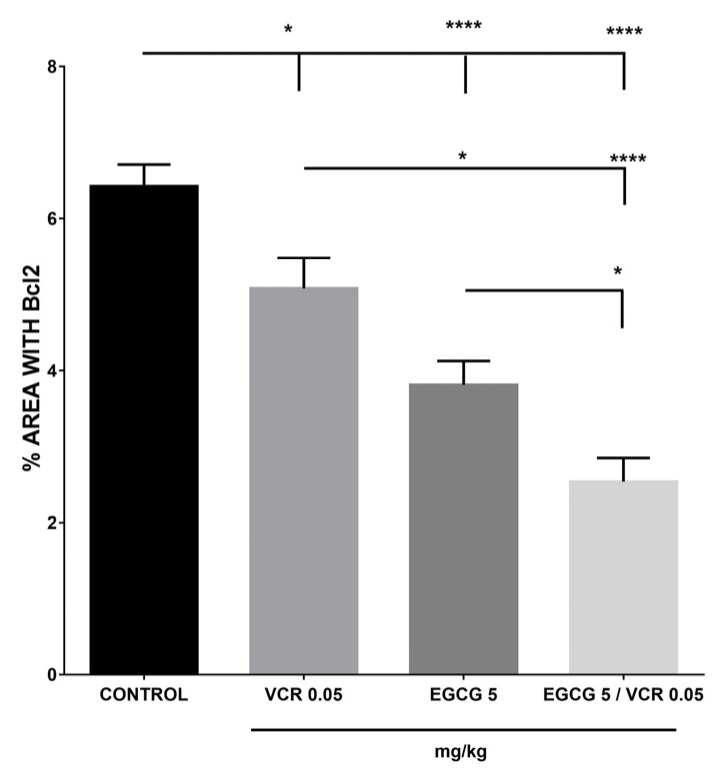
Effect of VCR, EGCG, and VCR/EGCG on the presence of Bcl2 in murine lymphoma. The results of immunopositivity with the Bcl2 protein showed that the control group presented the highest percentage in relation to the different treatments. A greater statistical difference was observed in the groups administered with EGCG and with the coadjuvant therapy of EGCG and VCR (*p* ≤ 0.0001). While with VCR the difference was *p* ≤ 0.05 in relation to the control, the decrease in Bcl2 immunopositivity presented among the groups with the different treatments, it was more noticeable with the coadjuvant treatment since it had a lower percentage compared to VCR and EGCG (*p* ≤ 0.05). NS is equal to no statistical difference; * and **** represent statistical difference of *p* ≤ 0.05 and *p* ≤ 0.0001 respectively. Each bar is the mean ± SE with n = 10. The data were analyzed by the statistical test of analysis of variance (ANOVA) followed by the comparison test multiple Tukey. Values of *p* < 0.05 were considered statistically significant.

Regarding the immunopositivity present in the different treatments with Bcl2. On one side, the control group was the one that had the highest percentage of labeling to the protein; in the other groups a significant decrease was observed in relation to the control in decreasing order VCR > EGCG >VCR/EGCG, with a significant difference between all treatments as seen in Figure 9.

## 3. Discussion

Despite the research and efforts made, so far, the treatments used in cancer have a very narrow therapeutic index and resistance. Among these drugs, vincristine is mostly used as a vital clinical agent for lymphomas, leukemias, and testicular cancer.

More alternatives can be examined to achieve less aggressive and more effective treatments. The effects of EGCG alone or combined with vincristine sulfate was observed in this study in a solid murine lymphoma model. The results mainly showed that while administering EGCG or VCR/EGCG, the tumor volume was smaller and the mice had a better life prognosis than the control group. An interesting finding was that the greatest inhibition in tumor development was with the VCR/EGCG group, significantly both VCR and EGCG and the combination presented an increase in p53 and a decrease in the expression of the antiapoptotic protein Bcl2, observing a more extensive decrease with concomitant treatment than with independent treatments.

An aggressive murine lymphoma was implemented to obtain these results which caused the death of 100% of the animals within 15 days. Among the antineoplastics to which it responds is vincristine sulfate [19].

It has been seen that this alkaloid acts in a dose-dependent manner [20]. In this study, only 0.30 mg/kg of vincristine presented smaller tumors and a better life expectancy than the control group, while 0.05 and 0.15 mg/kg showed no difference with said group. It has already been reported that at a dose of 0.15 mg/kg, no effect of vincristine was observed on survival [21]. Even though in antitumor therapy, the ideal is to use the highest possible dose to obtain the best result, with this alkaloid, the problem is that increasing the dose in a patient does not provide sufficient benefits in the benefit/toxicity balance; to the doses frequently used, the patient must accept some adverse reactions that do not justify the reduction of the dose, such as the first sensory changes [22] so it accepts that the treatment can lead to axonal degeneration [23], so it is significant to find substances that can increase the effect of this drug, to reduce the doses achieving the antitumor effect with fewer adverse reactions.

It is interesting that in a positive way within natural products it has been reported that the consumption of polyphenols contained in *Camellia sinensis* provides an antineoplastic benefit, among these phytochemicals there is an abundance of Epigallocatechin gallate (EGCG), which has an effect on different types of tumors in vitro and in vivo; however, importantly there is controversy in the results reported in vivo, which could be due to the model used, dose, and the low availability of EGCG in tumors [15].

In this model, EGCG showed a significant antineoplastic effect by negatively modulating the development of tumor volume and increasing survival in BALB/c mice with solid L5178Y tumor; among the different doses used, only the 5 mg/kg dose was significantly different from the control, obtaining 43% in size reduction, this is probably because the results showed high variability. Notably, the inhibition of tumor development by EGCG was similar to the effect obtained with the highest dose of vincristine used in this work (0.30 mg/kg).

To test the adjuvant effect of EGCG, the lowest dose (0.05 mg/kg) was used in this work. In this sense, when using both substances simultaneously, the antineoplastic effect was favorably different from the control and VCR alone, which did not happen when using only the alkaloid at this dose, and about survival. It was also higher when comparing these data with the result provided by EGCG in survival. The effect of the combination was similar to that provided by catechin alone, so it can be concluded that it comes from catechin and that vincristine does not interfere with said effect. The EGCG result with this dose coincides with that reported in a 4T1 study with breast cancer, who used 5, 10, and 20 mg/kg, with an inhibition of tumor growth in all administered doses of 20, 31, and 34% [24].

Unfortunately, there is a wide range of doses reported in the literature, for example, a delay in tumor development in a mouse lung cancer model mentioned with 10 mg/kg [25]; 25 mg/kg in breast cancer-positive to Estrogen Receptors [26] and in leukemia [27]; 30 mg/kg in osteosarcoma [28]; 50 mg/kg in triple negative breast [29]; 57 mg/kg in prostate carcinoma [30]; an effect has even been observed with doses as high as 100 mg/kg, as happened in bladder cancer where the tumor was reduced by 63% [31].

Therefore, the results so far are variable in terms of doses and the presence of the effect, which is not constantly observed as in the case of the investigation carried out with a xenograft of squamous cell carcinoma of the tongue in mouse [32], nor when using lung cancer A549 with 20 mg/kg EGCG [33].

In vitro results have also been controversial, attributed to the concentration, cell type, and aging of the cell culture [34].

The importance of finding the effect at a small dose (5 mg/kg) was that this dose was well below the tolerable oral dose of EGCG, which is 67.8 mg/kg daily for 14 days [35]. This dose also coincides with the administration in patients with breast cancer and radiotherapy (400 mg/day/8 weeks) where inhibition of the PI3K/Akt pathway was observed and arrested in G1 with a reduction of metastatic cells [36].

Inhibiting tumor growth with EGCG in this model results in the fact that tumor size is one of the main prognostic factors in cancer. As a result, volume reduction is one of the therapeutic strategies used, which may impact longer life. As was observed in this study, the groups with EGCG that reduced the volume had a higher probability of survival. These effects led to the belief that EGCG can be used as an adjuvant in order to improve the antineoplastic effect of vincristine. Various natural products have already provided favorable results when used concomitantly with another chemotherapeutics [37]. In the case of EGCG, an increase in the sensitivity to cisplatin was observed in ovarian cancer cells [38] and administered with taxanes, which contributed to inhibiting the growth of prostate tumor cells favoring their apoptosis through increasing p53, among other proteins [39], among other antineoplastics such as capecitabine [37], 5-fluorouracil, and doxorubicin [40].

In this sense, coinciding with this work, it is reported that the combination with vincristine had a favorable effect by inhibiting tumor growth in squamous cell carcinoma with VCR at doses of 0.46 mg/kg and EGCG 10, 20, and 40 mg/kg [41].

These results can be attributed to an increase in p53 since, in this work, the control group maintained low p53 positivity. This increased in the treated groups, with vincristine being the group that presented the highest immunopositivity to this protein and although VCR has related to the intervention in the mitotic process by binding to tubulin, preventing the assembly of microtubules and therefore the separation of chromosomes in metaphase, it has also been observed that it blocks cell proliferation by driving the cell to apoptosis. Importantly, VCR treatment has been reported to express p53 [42,43].

Therefore, the effect observed in this work can be partly due to the increase in the expression of p53. The increase in this protein was also present in EGCG, with this, it claims that EGCG causes cell cycle arrest and induces apoptosis by different mechanisms, including inhibiting angiogenesis, proliferation, migration, and metastasis. Among the different proposed pathways is the increase in the tumor suppressor p53, which is partly attributed to the inhibition of MDM2, the main negative regulator of p53, thus preventing its ubiquitination [44,45].

This increase in p53 was also observed in the group with the EGCG/VCR combination, without being different from the treatments independently; however, it is important that despite not being a summative effect, it continues to be present in the same proportion that means that the presence of p53 is not decreased through using both treatments concomitantly.

That is why the presence of said tumor suppressor is relevant, several actions have been attributed to it, including as a transcription factor that expresses several genes, is an inducer of apoptosis, favors genomic stability, and regulates the cell cycle concerning it is mentioned that stop the cycle in the G2 phase, but its main effect is in the G1 phase, in which it occurs mainly by the transcriptional activation of p21, with a decrease in Cyclin D1 and CDk4 and kinases. CDk6 and induces senescence in the cell and if the damage is not corrected, as a consequence, it causes apoptosis [46].

Cyclin D1, is produced by a proto-oncogene, mainly phosphorylates and inactivates the retinoblastoma (RB). It is known as an oncogenic protein because it is commonly overexpressed in cancer due to defective regulation at the post-translational level, increasing its presence and favoring the growth, cell cycle, and proliferation through various pathways, promoting angiogenesis and centrosome duplication in addition to functioning as a transcriptional modulator. Its relevance in cancer is such that it interacts with more than 100 proteins. All these functions mean that this oncoprotein can be used not only as a biomarker, but also as a therapeutic target for cancer, as an adjuvant therapy and as a targeted therapy [47]. But within the events of the cyclin mechanism, various points have also attracted attention as therapeutic targets. In this case, the inhibition of CDK4 and CDK6 has been sought. These are activated by Cyclin D1 to coordinate DNA replication and cell division, promoting proliferation, in this pathway the importance of its control points also stand out, the cyclin regulatory subunits. It is known that inhibiting the activity of cyclin-CDK complexes leads to inhibiting proliferation, among these inhibitors are INK4A (P16), P27 among others. One of the most studied is p21, which interacts with CDK, inhibiting its kinase activity, leading to cellular arrest, either by p53 or by other pathways, but p21 is also a transcriptional regulator, modulates apoptosis, and repairs DNA damage by interacting with proliferating. Cell nuclear antigen (PCNA) not only produces arrest in G1/S, but also in G2/M by inhibiting the CDK4-cyclin D, CDK6-CyclinD, and CDK2-Cyclin-E complexes [13,48,49].

It is mentioned that vincristine has a slight effect on the inhibition of Cyclin D1 but, it is not enough to stop the cycle in the G1-S phase [50].

On the other hand, it is reported that the blockage in the cell cycle provided by EGCG can be provided, among others, by strengthening the union between the CDK/CDKI and KIP1/p27 complexes with CDK2 and CDK4, of p27 kinase inhibitor proteins (KIP) as well as such as CDK inhibitors (INK) p16, p18, survivin, or p53. It can also regulate several proteins in the cell cycle, especially in the G0/G1 phase by decreasing Ciclyn D1 and increasing CIP/p21 through ERK, IKK, and PI3k. Interestingly, it was reported that in A431 skin cancer cells, EGCG did not alter the levels of p27, CDK2, and Cyclin D1 but did show inhibition of cell growth in vitro by increasing p21 and a decrease in CDK4 [48,51].

Similarly, in this work, no decrease in Cyclin D1 was observed in the tissues with any of the treatments used in this model, this leads to the assumption that the effect observed in the inhibition of tumor development was provided by another route other than the inhibition of Cyclin D1. It can be provided through the increase in p21 induced by p53 [48] or through apoptosis.

Due to the results obtained previously, in this work the effect of the different treatments on the Bcl2 protein was also observed. Since p53, in addition to delaying the cell cycle, leads to cells with damaged DNA to apoptosis, this process is normally regulated by an equilibrium between proapoptotic and antiapoptotic factors. By inhibiting antiapoptotic factors, the balance may tip to cell death. Among the antiapoptotic proteins, Bcl2 is distinguished. In this sense, p53 regulates the Bcl2 family through nuclear transcription, activating the expression of proapoptotic genes such as NOXA, Bax, and Puma, while in both the cytoplasm and mitochondrial membrane it inhibits the antiapoptotic Bcl2 and Bcl-xl, and activates proapoptotic proteins such as Bak and Bax, importantly, these proteins act through proapoptotic activators of BH3 to form pores in the outer mitochondrial membrane, releasing cytochrome C and favoring the activation of caspases [52]. The results show that both VCR and EGCG decreased the presence of said protein, but interestingly, the effect was greater in the VCR/EGCG combination, this was possibly because both substances decreased Bcl2. It is said that to prevent apoptosis, Bcl2 binds to Bax, preventing the permeabilization of the outer mitochondrial membrane and the release of cytochrome C, inhibiting apoptosis. Regarding VCR in breast cancer cells, this effect has already been described where Bcl2 hyperphosphorylation prevents its binding to Bax [53]. Furthermore, it reports that EGCG down-regulates the expression of Bcl2 mRNA [54]. The other mechanism described is due to an allosteric effect, to prevent the function of Bcl2 the gallate group binds with high affinity to the Bcl2 hydrophobic grooves selectively [55]. Therefore, Bcl2 reflects what the different mechanisms are for. One most likely induced through p53 acts in various stages of mitochondrial membrane permeabilization [56] and can be provided by both VCR and catechin and the second provided only by EGCG, which can explain the decrease in tumor volume and the increase in the survival prognosis observed.

## 4. Materials and Methods

### 4.1. Determination of Tumor Volume and Survival with (–)-EGCG, VCR, and Their Combination

To conduct this research project, the principle of the three R’s in animal experimentation was followed, for this reason, during this procedure, the fewest possible number of animals was used (5 for each group), which is supported by the experience of previous studies [19]. Only healthy mice BALB/c 10-week-old male were included in this study. These were purchased and maintained in the animal husbandry of the Autonomous University of the State of Hidalgo in an isolated room with an average temperature of 22 °C, humidity controlled, and light cycles of 12 h in polycarbonate boxes with a bed of sterilized and sieved sawdust, both food (Purina 308) and water were ad libitum. The tumor used in this work was murine lymphoma L5178Y TK+/- acquired in ATCC^®^ CRL-9518. This tumor is an ascitic fluid, and it was maintained by passages of 1×10^6^ intraperitoneal (IP) cells, every seven days [16]. The model used was a solid phase, with an adaptation period of 7 days; 1 × 10^6^ tumor cells were inoculated in the right gastrocnemius muscle to obtain a solid tumor [57]. The groups were made at random, occupying the boxes simultaneously.

Three days after this, the administration of the different treatments was carried out in the morning. For this purpose, 10 groups with 10 mice each, 5 individuals from each group, were used to determine tumor volume and five for the determination of survival evaluation. To avoid confusion, during the administration and results analysis stages, the groups were identified by color. Vehicles with distilled water received 0.1 mL intragastric route (IG); three groups received vincristine sulfate (Nefixol Ulsa Tech laboratory, Sinaloa, Mexico) in doses of 0.05, 0.15, and 0.30 mg/kg IP, every 7 days; three groups received EGCG [(–)-Epigallocatechin gallate^®^ from Sigma-Aldrich^®^ ≥ 95%; reference number E4143-50 mg] (COO MFCD00075940), in doses of 5, 25, and 50 mg/kg/24 h, (IG), dilution with distilled water was performed every day; three groups received the combination of the above treatments: VCR 0.05/EGCG 5 mg/kg; VCR 0.15/EGCG 5 mg/kg, and VCR 0.30/EGCG 5 mg/kg. Based on the previous results, the dose of 5 mg/kg was selected. To assess tumor volume, on day 11 post-inoculation, tumors were excised in 5 animals from each group. For this, the mice were anesthetized using the pre-anesthetic xylazine (24 mg/kg IM) and the dissociative anesthetic ketamine (80 mg/kg IP), after extraction. They were euthanized with sodium pentobarbital (150 mg/kg IP). Because the tumor developed in the gastrocnemius muscle, the tumor morphology showed a cone shape, so the volume was calculated using Formula (1). For determining the volume of a cone, the measurements in each of the tumors were performed ex vivo with a digital Vernier. Subsequently, the tumors were fixed with 4% formaldehyde. Survival was determined by recording the day of death of each individual in the different groups.

(1) $$V=\frac{1}{3}\pi r^{2}h$$

### 4.2. Determination of Proteins by Immunohistochemistry in L5178Y Tumor with (–)-EGCG, VCR and Their Combination

The already fixed tissues were dehydrated and impregnated in paraffin, 4 µm cuts were made. In performing immunohistochemistry for Cyclin D1, Bcl2, and P53, the sections were exposed to the antibodies Cyclin D1 sc-8396, Bcl2 sc-7382, and P53 sc-126 from Santa Cruz Biotechnology, INC; the contrast was performed with hematoxylin. For quantification of the immunopositive area, microphotographs of 10 fields of two tumors for each group were obtained: Control, VCR, EGCG, and the combination of VCR (0.05 mg/kg) and EGCG (5 mg/kg) with the 10× objective. A Carl Zeiss microscope was used, and photographs were taken with a Celestron digital microscope camera. The images were analyzed with the Imagen J software version 2.0.0-rc-69/1.52p to quantify the percentage of positivity of the different proteins in the tissue, and the results were expressed in micrometers (μm) [58,59,60].

The handling and care of the animals were executed by trained veterinarians and following the Guide for the care and use of laboratory animals of the Committee of the National Research Council (USA-US), as well as the Mexican NOM Standards. -062-ZOO-1999, the Internal Ethics Committee approved the project for the Care and Use of Laboratory Animals (CIECUAL) of the UAEH with the number CIECUAL/007/2019.

### 4.3. Statistical Analysis

Expressing the results with descriptive statistics as the mean ± SEM. The data were analyzed by the statistical test of analysis of variance (ANOVA), followed by the Tukey multiple comparison test. The data were processed through the Kaplan–Meier, Log-Rank survival analysis, and all analyses were performed with GraphPad Prism 7.0. All analyses were performed with GraphPad Prism 7.0. Values of *p* < 0.05 were considered statistically significant.

## 5. Conclusions

In this work, the antitumor activity was observed with EGCG and the adjuvant treatment of VCR/EGCG, by inhibiting tumor growth and increasing survival, possibly through increasing p53 and decreasing Bcl2, observing a greater effect in the concomitant treatment VCR/EGCG for Bcl2. The VCR/EGCG combination was more effective in inhibiting tumor development than the independent treatments.

## Data Availability

The data generated in this work are available to those interested when requested.
